# Hyoid Bone Fracture Pattern Assessment in the Forensic Field: The Importance of Post Mortem Radiological Imaging

**DOI:** 10.3390/diagnostics14070674

**Published:** 2024-03-22

**Authors:** Vincenzo Cianci, Cristina Mondello, Annalisa Cracò, Alessio Cianci, Antonio Bottari, Patrizia Gualniera, Michele Gaeta, Alessio Asmundo, Daniela Sapienza

**Affiliations:** 1 Department of Biomedical and Dental Sciences and Morphofunctional Imaging, Section of Legal Medicine, University of Messina, via Consolare Valeria, 1, 98125 Messina, Italy; mondelloc@unime.it (C.M.); patrizia.gualniera@unime.it (P.G.); alessio.asmundo@unime.it (A.A.); 2 Diagnostic and Interventional Radiology Unit, Department of Biomedical Sciences and Morphological and Functional Imaging, University Hospital Messina, 98125 Messina, Italy; annalisacraco@hotmail.it (A.C.); bottaria@unime.it (A.B.); mgaeta@unime.it (M.G.); 3 Department of Cardiovascular Medicine, Fondazione Policlinico Universitario A. Gemelli-IRCCS, Largo A. Gemelli 8, 00168 Rome, Italy; alessiocianci.1998@gmail.com

**Keywords:** manual strangulation, charred corpse, forensic pathology, PMCT, post mortem CT, hyoid bone fracture

## Abstract

Post mortem hyoid bone fracture findings may be attributable to various factors, including both the onset of acute mechanical asphyxia as it happens in manual strangulation and in charred corpses. In forensic practice, the discovery of corpses burned after death to hide their real cause of death is not uncommon: in these cases, the diagnostic challenge is even greater, as the action of flames is capable of both masking previously generated lesions and/or generating new ones, as occurs for hyoid bone fractures. The case concerns a 76-year-old man found charred in his bedroom. Almost complete body charring made it impossible to evaluate any external damage. Post mortem computed tomography (PMCT) was performed, and an evident bilateral fracture of the greater horn of the hyoid bone was detected. Although the absence of typical charring signs had steered the diagnosis towards post mortem exposure to flames, PMCT proved to be very useful in increasing the accuracy in correctly determining the cause of death. In particular, making use of Maximum Intensity Projection (MIP) hyoid bone reconstructions, it was possible to measure the medial dislocation angle of the fracture fragments and then to establish the applied direction of force, which acted in a lateral–medial way. A manual strangulation diagnosis was confirmed. The increasing importance of performing post mortem radiological exams as a corollary for conventional autopsy has been further confirmed.

## 1. Introduction

Hyoid bone fracture identification during an autopsy examination frequently suggests that compressive forces may have been applied to the neck region [1]. It is known that during the occurrence of some acute mechanical asphyxia syndromes, such as ligature strangulation and manual strangulation, the finding of such fractures can be considered relatively common [1]. Furthermore, this type of lesion can also be detected in charred corpses [2].

Manual strangulation falls within acute asphyxia syndromes, representing one of its five subtypes, along with hanging, ligature strangulation, suffocation, and drowning [3]. In suffocation, obstruction of the air passage occurs due to compression of the external respiratory orifices, whereas in drowning, it occurs due to the presence of liquid components that replace the interalveolar air [4]. In hanging and ligature and manual strangulation, asphyxia occurs due to the neck region’s compression, leading to air passage impairment [3]. In hanging, the force that causes the lace constriction is represented by the body’s weight itself, and hyoid bone fracture is described as less common in this case [5]. In ligature strangulation, the airway’s occlusion is secondary to constriction of a lace around the neck, whereas in manual strangulation, the offender uses his/her own hand, arm (chokehold), or leg to put pressure on the neck: in these cases, hyoid bone fracture is described as more common [6,7].

Hyoid bone fracture may also be a consequence of charring [8]. Charred corpses represent a challenge for forensic pathologists, as the heat generated by the fire can modify the common cadaveric findings usually analyzed during inspection and autopsy examination. The action of flames is capable of both masking the injuries inflicted on the body before its exposure to the flames and/or generating new ones: among the latter, some are typical of other conditions, such as hyoid bone and/or cranial fractures (sign of diploe) up to the occurrence of limb amputations, which makes reconstruction of the dynamics that led to death even more complex [8]. Therefore, in forensic practice, it is of great importance to distinguish between heat-related lesions and those that are trauma-related, as well as ante mortem and post mortem injuries [8]. Thermal amputations usually appear as transverse fractures with smooth margins, uncovered by soft tissues, as the cortices are directly exposed to the flames (a so-called “flute-mouthpiece” appearance); on the other hand, traumatic fractures are mostly covered by soft tissues and show clean, angulated margins, frequently associated with evident comminution [8].

In the literature, several attempts to mask the real dynamics that led to death have been described: murderers may try to burn the bodies of people already deceased, both aiming to hide them and make personal recognition more complex [9]. Therefore, it is first necessary to look for signs that allow us to distinguish between deaths due to charring and the charring of corpses [9]. Among these, toxicological examination to evaluate the carboxyhemoglobin percentage and soot findings in the upper and lower airways are considered the most relevant ones [8].

It should also be underlined that the exposition of bodies to flames during carbonization processes usually leads to muscle and tendon structure dehydration. This phenomenon is responsible for the appearance of the so-called “boxer posture”, characterized by the flexed attitude of the limbs, which is reminiscent of a fighter pose [10]. Muscle fiber dehydration and contraction are not exclusively limited to the limbs but involve all body segments, including those muscles inserted onto the hyoid bone: the traction exerted by their progressive contraction can produce a hyoid bone fracture itself, which usually occurs at the greater and lesser horns, presenting characteristics similar to those of manual strangulation.

## 2. Case Report

**Figure 1:**
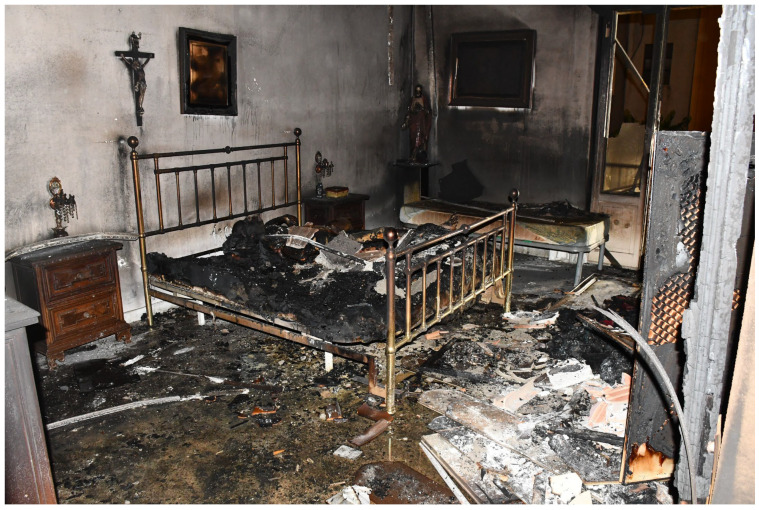
Charred body found at the crime scene.

This case concerns a 76-year-old man found charred in his bedroom, lying on his bed. The firefighters had taken steps to put out the flames, which had broken out and only affected the bedroom but no other rooms (Figure 1). A shattered glass relief on the floor, near a balcony overlooking the internal cloister of the house, was reported. The flat was located on the first floor of the building.

Although a few hours had passed since the fire was resolved, a strong petrol smell was still perceptible inside the house. The presence of an accelerant (fuel) was then documented by the firefighters, supporting a fraudulent hypothesis behind the fire itself.

**Figure 2:**
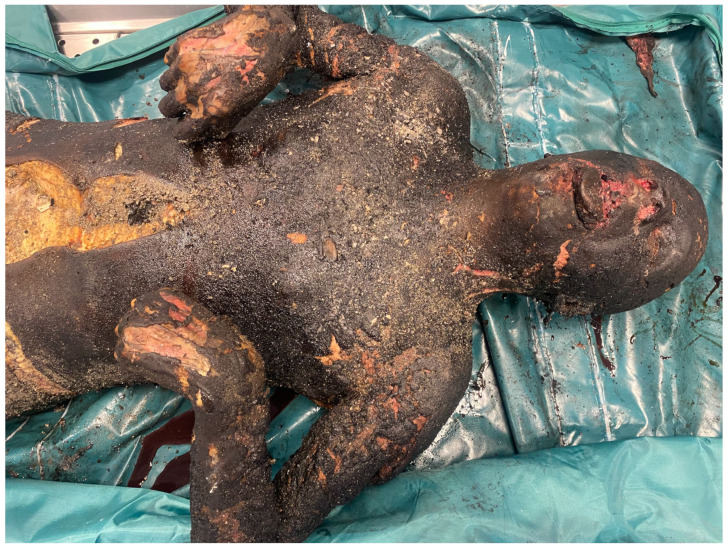
Boxer posture of the charred corpse.

The alleged homeowner’s body lay supine on the bed, being almost completely charred, except for the distal right lower-limb tract, which appeared to have been slightly spared by the flames. Upon inspection, the corpse presented a typical flexed attitude in the limbs, which is better known as a “boxer posture” (Figure 2). It is important to underline that the body’s dorsal region, which was in contact with the bed, was not damaged by the flames: therefore, it is reasonable to assume that during the flames’ action, the subject remained immobile, despite not having any movement limitations. Furthermore, due to the charring, it was not possible to acquire any supplementary data that would have definitively allowed for personal identification of the victim. On behalf of the judicial authority, a post mortem computed tomography (PMCT) examination and autopsy were performed.

**Figure 3:**
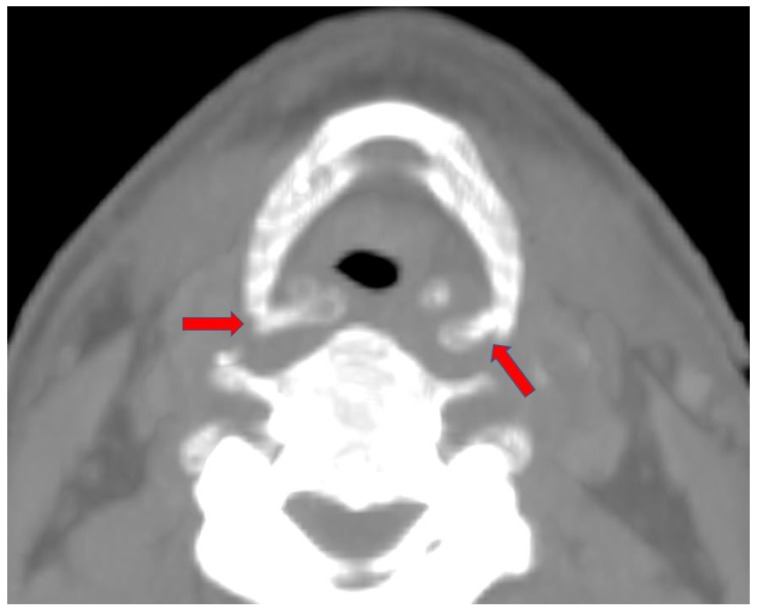
An axial MIP CT image demonstrates the bilateral fracture of the greater horn of the hyoid bone (red arrows).

The PMCT radiological examination highlighted an evident bilateral fracture of the greater horn of the hyoid bone. Maximum Intensity Projection (MIP) reconstructions of the hyoid bone itself were subsequently performed (Figure 3).

**Figure 4:**
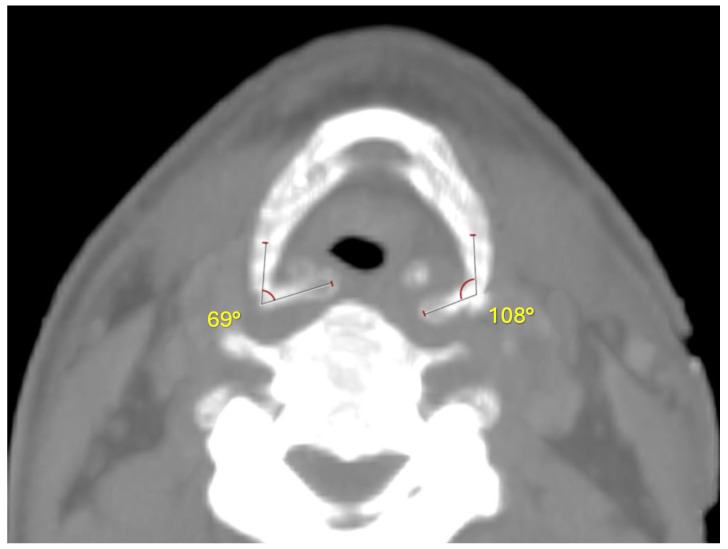
Measurement of the medial dislocation angle of the fracture fragments, compared with their normal sagittal position.

**Figure 5:**
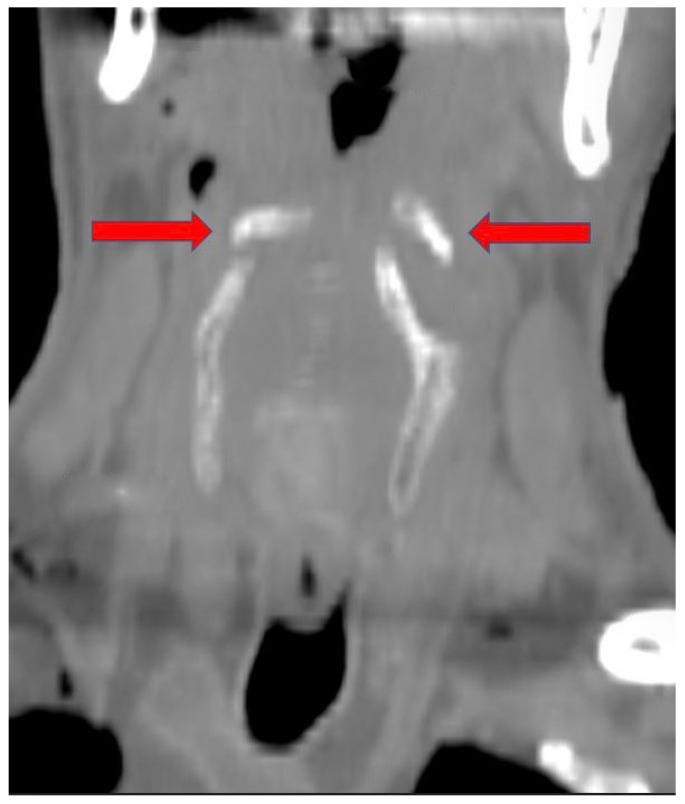
Coronal CT image shows that the dislocation of the two fragments can only be explained by the action of forces acting in the lateral–medial direction (red arrows).

It should be highlighted that the two fracture stumps are displaced medially and horizontally, with a more evident dislocation on the right side than on the left one (Figure 4 and Figure 5). The type of dislocation observed can be considered a consequence of the application of force vectors acting in the lateral–medial direction.

An autopsy was performed, and the main macroscopic and microscopic findings were analyzed. Due to the charring, no diagnostic findings were obtained from external inspection of the corpse, not even in the neck region (Figure 4).

However, after the skin plane’s detachment, it was possible to detect a large area of hemorrhagic infiltration in both the superficial and deep neck muscles and the peri-laryngeal soft tissues. The occurrence of a hyoid bone fracture was also confirmed.

After the neck organs’ removal, the main characteristics of the respiratory tract’s mucosa were observed, trying to evaluate the presence of soot deposits. A small quantity of soot was found in the buccal cavity, while its complete absence not only in the lower airways but also in the more proximal regions of the tracheal mucosa was described. After carrying out the histological examination, no evidence of thermal injuries at the alveolar–capillary level was detected. Both hyperaerated and ecstatic alveolar spaces—probably due to the interruption of the septa—suggestive of acute pulmonary emphysema and plurivisceral congestion were reported.

Toxicological analysis of the peripheral venous blood to quantify the carboxyhemoglobin levels was also performed: a carboxyhemoglobin value of 3% was found. Both the absence of soot in the airways and the finding of carboxyhemoglobin values within the normal ranges were considered fundamental data for excluding the hypothesis of death related to the flames’ action. In fact, the negativity of these two signs can be considered representative of the subject’s lack of respiratory activity during flame exposure. Equally suggestive are both the absence of thermal lesions in the distal airways and heat lesions in the dorsal region of the body, probably due to its immobility during flame exposure. The hyoid bone fracture finding, the occurrence of which can be related to fire dehydration phenomena, was traced back to manual strangulation thanks to stump fracture pattern reconstruction using PMCT. Manual strangulation was therefore identified as the cause of death.

In the following days, the police arrested the criminal, who confessed to the murder and the subsequent attempt to hide it by setting the fire.

## 3. Discussion

The reported case highlights the importance of using post mortem radiological techniques as integrative autopsy methods, useful for corroborating a medico-legal diagnosis of death. In particular, the importance of PMCT in differential diagnosis between the causes that can determine a hyoid bone fracture has been pointed out. PMCT can then be considered a particularly useful tool for correctly identifying the mechanism that determined the hyoid bone fracture in subjects who have died of manual strangulation and subsequently been burned [11,12].

It is also important to underline the possibility of using other radiological methods, such as the X-ray method (RTG): RGT images allow us to evaluate the internal structure of an examined body part and therefore to identify injuries that may have occurred [13]. Despite this, it is known that PMCT can lead to the acquisition of three-dimensional images, and it therefore allows us to conduct much more accurate spatial analyses [14].

The hyoid bone is a median unpaired bone, and it is not articulated with the other skull bones [12]. It is located on the fourth cervical vertebra plane, antero-superiorly to the esophagus, below the mandible, and above the larynx [12,15]. It is a mobile bone, being the insertion site of several muscles, such as the suprahyoid and infrahyoid muscles [16]. It consists of a body and four appendages, the so-called greater and lesser horns [12]. It is important to highlight that among the muscles inserting into the hyoid bone, the mylohyoid and styloid muscles are those inserting superiorly, whereas the thyrohyoid muscles insert inferiorly [17]. The styloid ligament is inserted into the lesser horns, connecting it to the styloid process of the temporal bone [17]. Lastly, the greater horns, which have a medial concavity and point upwards and backwards, represent the insertion site of the hyoglossus, thyrohyoid, and middle pharyngeal constrictor muscles [17].

As mentioned before, flame-induced dehydration phenomena can lead to muscle contraction, which is then capable of producing a hyoid bone fracture because of the occurrence of dehydration phenomena [11].

In fact, the action of flames on corpses, especially if high temperatures are reached, is considered sufficient to determine the rapid dehydration of the whole body’s muscular and tendon structures [11]. As a direct consequence of the dehydration phenomena, the upper and lower limbs contracting in their attitude, which is better known as a “boxer posture”, can be frequently detected [10]. Temperatures between 670 °C and 810 °C are considered sufficient to produce a “boxer posture” after 10 min of continuous exposure [10].

Regardless, to better understand the mechanisms leading to a hyoid bone fracture in charred bodies, first, the extreme mobility of the hyoid bone itself should be considered, which is physiologically capable of adapting to muscular contraction and relaxation [18]. Despite this ability, it is reported that if extreme contracture of the hyoglossus and middle constrictor muscles occurs, this is capable of determining the fracture of the upper greater hyoid bone horns [11,18]. In fact, the hyoglossus and middle constrictor muscles exert vectors of force in the caudo-cranial direction, potentially determining the greater upper horns’ fracture, with an upward dislocation of the stumps [11,19].

Conversely, medial stump displacement indicates the horizontal application of force vectors, as happens in manual strangulation [20]. However, sole evaluation of the dislocation patterns of the hyoid bone fracture stumps cannot be considered sufficient to attribute the fracture itself to the carbonization process or manual strangulation.

Therefore, the correct diagnostic process for the right diagnostic assessment of charred corpses must include analysis of those factors representative of the subject’s vitality during flame exposure [21]. Among these, the presence of soot in the respiratory tract is considered representative of a subject’s vitality during flame exposure; in fact, due to the presence of respiratory activity, carbonaceous particles are inhaled and subsequently observed during autopsy examination of the respiratory tract’s mucosa, beyond the bifurcation of the large bronchi [22]. Signs of thermal injuries can also be detected [23].

Likewise, both inhalation of the gases produced during combustion processes and maintenance of alveolar–capillary gas exchange during exposure to flames are responsible for the increase in the percentage of carboxyhemoglobin in the blood. It is known that the percentage of carboxyhemoglobin detectable in healthy subjects is usually less than 2%. Despite this, percentages of up to 10% can be found in “heavy smokers” [24]. In the literature, carboxyhemoglobin values above 10% are considered consistent with inhalation of smoke from a fire [25].

Other studies have reported carboxyhemoglobin values up to 10–20% in heavy smokers; therefore, some authors do not consider values within these ranges sufficient to make a diagnosis of viability in charred bodies [26].

Wirthwein D. P. et al. [27] have proposed carboxyhemoglobin values >30% to be strongly suggestive of combustion product inhalation during flame exposure, whereas a level of <20% should prompt a search for other causes.

In manual strangulation, one of the most suggestive diagnostic signs is represented by the characteristic type of bruises reproducing an attacker’s fingertips, usually described in the antero-lateral neck region and frequently associated with clawing [25]. Hemorrhagic infiltration of the neck muscles and hyoid bone fracture can be frequently observed [28].

Furthermore, signs of a struggle and defensive injuries in other areas of the skin on the victim’s body can be detected. On histological examination, typical signs are represented by the presence of hyperaerated and ecstatic alveolar spaces, visceral pleura congestion, and hemorrhagic infiltration of the parathyroid and laryngeal cartilage and soft tissues [29].

In the described case, the almost complete charring of the corpse did not allow for observation of the external signs typical of manual strangulation, and further examinations were conducted. PMCT led to the identification of the hyoid bone fracture and subsequent analyses of the stump dislocation. Furthermore, both the negativity of soot in the lower airways and the detection of carboxyhemoglobin values within the normal range (<10%), as well as hemorrhagic infiltration of the neck muscles and soft tissues and the histological signs of acute pulmonary emphysema, oriented the diagnosis towards manual strangulation.

## 4. Conclusions

The use of post mortem radiological methods is increasingly widespread worldwide: among these, PMCT is certainly the most used. PMCT is usually considered a corollary to autopsy, but in certain conditions, it can provide information not elsewhere available. In the described case, both the absence of soot and thermal lesions in the distal airways and the finding of carboxyhemoglobin values lower than 10% made it possible to time the death to before the exposure of the corpse to the flames. The almost complete charring of the corpse did not make it possible to identify external signs but only internal signs of manual strangulation in the structures underlying the skin’s surface, such as the hyoid bone fracture, allowing a medico-legal diagnosis to be made.

Despite this, the possibility of obtaining both the direction and dislocation angles of the fracture stumps through PMCT reconstruction allowed for the diagnosis of manual strangulation to be confirmed and strengthened, significantly increasing the diagnostic precision.

## Data Availability

All the data are reported in the paper.
